# Periodontitis Stage III–IV, Grade C and Correlated Factors: A Histomorphometric Study

**DOI:** 10.3390/biomedicines7020043

**Published:** 2019-06-12

**Authors:** Barbara Buffoli, Gianluca Garzetti, Stefano Calza, Eleonora Scotti, Elisa Borsani, Veronica Cappa, Lia Rimondini, Magda Mensi

**Affiliations:** 1 Division of Anatomy and Physiopathology, Department of Clinical and Experimental Sciences, University of Brescia, 25123 Brescia, Italy; elisa.borsani@unibs.it; 2 Section of Periodontics, School of Dentistry, Department of Surgical Specialties, Radiological Science and Public Health, University of Brescia, 25123 Brescia, Italy; gianluca.garzetti@gmail.com (G.G.); eleo.scotti@gmail.com (E.S.); magda.mensi@unibs.it (M.M.); 3 Department of Molecular and Translational Medicine, University of Brescia, 25123 Brescia, Italy; stefano.calza@unibs.it (S.C.); veronica.cappa@unibs.it (V.C.); 4 Big & Open Data Innovation Laboratory (BODaI-Lab), University of Brescia, 25123 Brescia, Italy; 5 Department of Health Sciences, University of Piemonte Orientale “UPO”, 28100 Novara, Italy; lia.rimondini@med.uniupo.it; 6 Center for Translational Research on Autoimmune & Allergic Diseases—CAAD, University of Piemonte Orientale “UPO”, 28100 Novara, Italy

**Keywords:** periodontitis, histomorphometric analysis, smoke, plaque, aging

## Abstract

Background: Periodontitis is a disease that leads to serious functional and esthetic dysfunctions. Periodontitis exists in different forms, and its etiology is related to multiple component causes. Two key processes involved in the evolution of this pathology are angiogenesis and inflammatory infiltrate. The aim of this study was to understand if important factors such as smoking, gender, age, plaque, pus, and probing pocket depth could influence the histomorphological pattern of generalized stage III–IV, grade C periodontitis (GPIII–IVC), which is a particular form of periodontitis. Methods: Eighteen subjects with GPIII–IVC were enrolled in this study. The percentage of inflammatory cells and the vascular area were measured and evaluated in relation to each periodontal disease-associated factor. Results: Females showed a significant increase in the percentage of inflammatory cells compared to males (6.29% vs. 2.28%, *p*-value = 0.020) and it was higher in non-smokers than in smokers (4.56% vs. 3.14%, *p*-value = 0.048). Young patients showed a significant increase in vascular area percentage compared to older patients (0.60% vs. 0.46%, *p*-value = 0.0006) and this percentage was also higher in non-smokers compared to smokers (0.41% vs. 0.55%, *p*-value = 0.0008). The vascular area was also more than halved in subjects with residual plaque on tooth surfaces (0.74% vs. 0.36%, *p*-value = 0.0005). Conclusions: These results suggested that even if these factors are commonly related to the worsening of periodontal status, some of them (pus and periodontal probing depth (PPD)) do not affect the inflammatory and vascular patterns.

## 1. Introduction

Periodontitis is a disease that, if untreated, leads to serious functional and aesthetic impairments, as well as a strong conditioning of social life, resulting in impairment in life quality [1].

This disease is characterized by a microbially-associated, host-mediated inflammation that results in the loss of periodontal attachment, clinically detected as clinical attachment loss (CAL) [2]. If not treated, periodontitis can result in impaired occlusion, occasional pain, discomfort, and, eventually, tooth loss [3].

In 2018, the American Academy of Periodontology and the European Federation of Periodontology published a new classification of periodontal diseases. Here is shown a periodontitis case definition system based on a staging and grading framework. While stages I to IV are defined based on the severity and complexity of management, grades A to C evidence the disease progression rate in three categories: slow, moderate, and rapid. Depending on disease distribution and extent, periodontitis can be categorized into a localized (<30% of teeth involved) generalized or molar/incisor pattern [4].

The worst periodontal condition is evident in patients with generalized stage III–IV, grade C periodontitis. In these situations, significantly rapid progressive damage to the attachment apparatus, which can lead (especially in stage IV) to tooth loss and occlusion impairment, is appreciated [4].

About the etiology of this pathology, periodontitis is a complex disease with a genetic and epigenetic basis and/or causes related to patient behaviors (e.g., medications or environmental factors), which contribute to the progression of the periodontal lesion. In addition to such ‘patient-specific risk factors,’ there are also ‘site-specific characteristics,’ such as anatomical factors, which may promote the development of a lesion [1].

Periodontal lesions induce tissue changes inside the gum and the alveolar bone. The constant action of the etiological factors, which induces and sustains pathological changes, can induce irreversible changes. Angiogenesis together with inflammatory infiltrate are associated with the evolution of gingival inflammatory processes [5,6]. During gingival inflammation, inflammatory infiltrates represent an increasing part within the connective tissue causing collagen degradation or fibrotic reaction [7,8] by stimulating the effects of inflammatory mediators of the connective tissue [9,10]. In addition, inflammation is associated with pathological angiogenesis and a high number of newly formed blood vessels that can be quantified as micro-vessel density (MVD) [6,7,8,9,10,11].

Considering the involvement of these two processes in GPIII–IVC pathogenesis, the aim of our study was to evaluate these histomorphological alterations in relation to some important factors (e.g., smoking, gender, age, plaque, pus, and PPD (probing pocket depth)), known as periodontal disease-associated factors. In particular, our null hypothesis had been to find a statistically significant major mean percentage of the vascular area and inflammatory cells in non-smoker patients due to the effects of smoking on biological tissues. GPIII–IVC patients have been chosen to better define the histomorphological characteristics of a low prevalence illness.

## 2. Materials and Methods

### 2.1. Study Design and Ethical Approval

This retrospective observational study was conducted at the Dental Clinic “Lidia Verza,” University of Brescia, Italy, from January 2014 to November 2016. The study protocol was reviewed and approved by the ethics committee of “AO Spedali Civili” Hospital of Brescia (protocol n° 1473) (0059683, 18/12/2013) and conducted in accordance with the Helsinki Declaration. Written informed consent was obtained from all patients.

### 2.2. Patients and Collection of the Samples

Eighteen subjects were enrolled in this study. The inclusion criteria were age older than 18 years; no systemic illnesses or disorders; no medical treatment that may impair healing (immunodepression, immunosuppression, diabetes, etc.); diagnosis of GPIII–IVC [4]; the presence of at least five teeth in each quadrant; and the ability and willingness to give written informed consent.

The exclusion criteria included pregnant or breastfeeding women; women practicing birth control methods; cancer; allergy or other severe adverse reactions to amoxicillin and metronidazole; use of local and/or systemic antibiotics in the 6 months previous to the beginning of the study.

Age, gender, smoking habits, and clinical and dental history were collected during clinical assessments. Pocket probing depth (PPD), clinical attachment level (CAL), gingival margin recession (GMR), bleeding on probing (BoP), and the plaque index (PI) using a periodontal probe with a diameter of 0.5 millimeters (UNC 15, Hu-Friedy Italy, Milan, Italy) were evaluated.

The collection of the samples was carried out under topical anesthesia with 2% mepivacaine and epinephrine 1:100,000 [7,8].

Hopeless [12] and mono- and multi-rooted teeth were selected (intra-radicular tissue was not considered). Clinical data were collected, and six sites for teeth were tested. Gingival biopsies (2 mm × 2 mm) from the attached tissue around a selected natural tooth were taken by scalpel (15C, Hu-Friedy Italy, Milan, Italy) from the gingival margin of the deepest sites (PPD ≥5 mm). Tissue samples were fixed in 10% buffered formalin (pH 7.0–7.2) for 24 hours, dehydrated, and embedded in paraffin according to standard procedures. Serial sections (7-μm thick) were cut by a microtome (Microm HM 325, Thermo Scientific, Walldorf, Germany) and collected on poly-l-lysine-coated glass slides.

### 2.3. Histomorphometric Analysis

**Figure 1:**
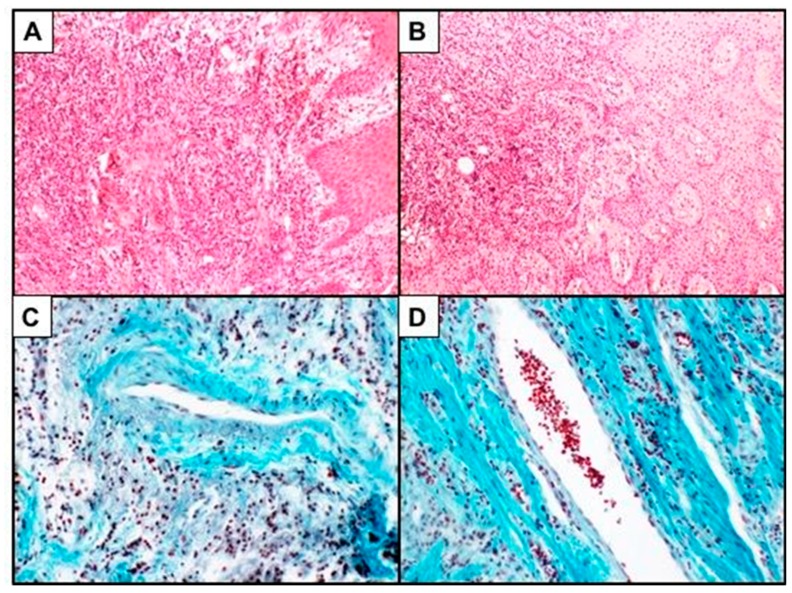
(**A**,**B**): Hematoxylin and eosin staining (×100) showing inflammatory cells in the gingival mucosa; (**C**,**D**): Masson–Goldner trichrome staining showing vessels in the gingival mucosa (×200).

Sections were deparaffinized in xylene, rehydrated, and stained with hematoxylin–eosin staining (Bio-Optica, Milan, Italy) and Masson–Goldner trichrome staining (Merck KGaA, Darmstadt, Germany) for the evaluation of the percentage of inflammatory cells and the vascular area (Figure 1).

In order to quantify the percentage of inflammatory cells and the percentage of the vascular area, digitally fixed images (arbitrary standardized area) for each section (five serial sections/sample) were analyzed by an optical light microscope (Olympus BX50, Olympus, Hamburg, Germany). Histomorphometric analyses were performed by two blinded investigators at the Section of Anatomy and Physiopathology of the University of Brescia. Quantitative analysis of the percentage of inflammatory cells and the vascular area were performed using a camera equipped with an image analysis system (Image-Pro Premier 9.1; Immagini e Computer, Milan, Italy).

### 2.4. Statistical Analysis

Statistical analyses were performed by statistician from the University of Brescia.

As the percentage of non-smokers who generally attend the clinic is about 60%, the sample size was calculated assuming a 2 vs. 3 ratio between arms (smokers vs. non-smokers). A sample size of 18 subjects (11 non-smokers and 7 smokers) is sufficient to detect a clinically important difference of 0.45% between groups in reducing the vascular area, assuming a mean in healthy and non-smoking patients of 1% [13], a common standard deviation of 0.3% with 80% power, and a 5% level of significance.

Similarly, as the mean of inflammatory infiltrates reported by literature in healthy and non-smoking patients is 9% with a standard deviation of 1.5% [1], assuming a 2.5% reduction in smokers (mean 6%), a sample size of 16 patients (10 non-smokers and 6 smokers) would deliver an 80% power at a 5% level of significance.

In order to consider both aims, 18 patients were enrolled in this study.

The binary coded variable BoP was summarized as counts and percentages. Continuous variables such as PPD and CAL loss were summarized as geometric mean and standard deviation (sd). Due to the high number of zeros, the gingival recession was summarized both as the percentage of zero values and the geometric mean and sd of non-zero values.

Laboratory data on inflammatory infiltrates and alveolar areas were recorded as within patient averages and standard deviations; therefore, statistical analyses accounted for measurement precision using inverse variance weighting. We used univariate linear regression models to evaluate the relationship between log-transformed outcome variables (inflammatory infiltrate and vascular area) and clinical determinants: gender (male/female), age (coded as <51 and ≥ 51 years old), smoking habit (smoker/non-smoker), PPD (coded as <9 mm and ≥9 mm), presence of plaque on tooth surface (yes/no), and pus (yes/no). The results are reported as the estimates and 95% confidence intervals. All analyses were performed using the statistical software R (version 3.5.2, https://www.R-project.org/.) with a significance level of 5%. The sample size was computed using G*Power (version 3.1, http://www.gpower.hhu.de/).

## 3. Results

Out of 18 patients, 72% were females, 55.6% were more than 51 years old and smokers. About two-thirds of the subjects had plaque on the tooth surface and pus. Full mouth means of PPD, CAL, GMR (different from 0), and BoP were 3.46 mm, 4.03 mm, 2.39 mm, and 49%, respectively (Table 1).

Females showed a significant increase in inflammatory infiltrate compared to males (6.29% vs. 2.28%, *p*-value = 0.020) and this increase was also higher in non-smokers than in smokers (4.56% vs. 3.14%, *p*-value = 0.048).

Considering the vascular area, young patients showed a significant increase compared to older patients (0.60% vs. 0.46%, *p*-value = 0.0006) and this increase was also higher in non-smokers than smokers (0.41% vs. 0.55%, *p*-value = 0.0008). The vascular area was also more than halved in subjects with residual plaque on the tooth surfaces (0.74% vs. 0.36%, *p*-value = 0.0005), see Table 2.

## 4. Discussion

The present retrospective observational study aimed to understand the influence of smoking, gender, age, plaque, pus, and probing pocket depth on the histomorphological pattern of GPIII–IVC.

Females showed a significant increase in inflammatory infiltrate compare to males (and it was higher in non-smokers than in smokers). Considering the vascular area, young patients showed a significant increase compared to older patients and it was higher in non-smokers than smokers. Our results showed that the vascular area was also more than halved in subjects with residual plaque on tooth surfaces.

Periodontal disease and its related risk factors have been studied with growing interest [14,15,16]. This increasing investigation is due to the important scientific evidence that shows a variety of systemic diseases and conditions that can affect the course of periodontitis or have a negative impact on the periodontium (e.g., genetic disorders, acquired disorders, immunodeficiency and inflammatory diseases) [17]. In addition, a recent study has pointed out that periodontitis is strongly associated with oral squamous cell carcinoma (OSCC) and subjects with periodontitis are 3.7 times more likely to have OSCC than subjects without periodontitis [18]. An abnormal inflammatory response to some periodontal pathogenic bacteria belonging to the red complex (e.g., *Tannerella Forsythia*) including pro-inflammatory cytokines (IL-1β and IL-6) overexpressed by CD4+ T helper cells and TNF-α, and the presence of other molecules such as gingipain K produced by *Porphyromonas gingivalis* affecting the host’s immune system by the degradation of immunoglobulins and the complement system (C3 and C5 components), are suggested as the base of the increased risk of head and neck cancer development [19], as well as the occurrence of the same lifestyle-related risk factors including tobacco smoking.

With this assumption, we decided to discuss the results factor by factor for simplifying the comprehension.

### 4.1. Gender

Women showed a statistically significantly higher percentage of inflammatory cells with respect to men and a non-significant reduction in the percentage of the vascular area. In the literature, gender differences in periodontal diseases have been reported. The authors affirmed that men seemed to be more susceptible to the risk of periodontal disease than women; on the contrary, men did not show a higher risk of more rapid periodontal destruction than women [20]. This difference could be considered to understand our results. However, additional studies aimed at evaluating the incidence of gender in the characterization of the vascular and inflammatory patterns of periodontal disease should be conducted.

### 4.2. Age

Our results showed a significant decrease in the percentage of the vascular area in patients older than 50 years. These data are in accordance with Preshaw et al. [21] who asserted that the immune system in old age is compromised by physiological changes, where there is a concomitant decrease in blood vessel density with increasing age.

### 4.3. Smoking

Tobacco and its oxidation products are involved in the progression and clinical alterations of periodontal diseases. Prakash et al. [21] reported a significant decrease in microvascular density and inflammatory cell percentage in smokers with periodontitis compared to non-smokers. According to the literature, our results confirm a significant decrease in the percentage of the vascular area and the inflammatory cell percentage in smoker patients affected by GPIII–IVC [21,22].

### 4.4. Pocket Probing Depth

A periodontal pocket is defined as a pathologically deepened gingiva sulcus, according to the definition reported by the American Academy of Periodontology. It occurs with the destruction of the supporting periodontal tissue and migration of alveolar bone, periosteum, and periodontal ligament versus the tooth apex.

The vascular density of the marginal gingiva is supported by arteries that extend into the periodontal ligament and the alveolar bone and periosteum [23]. Consequently, we can speculate that vascularization of the marginal gingiva should be more altered in relation to the pocket depth. This concept is supported by our results, which showed a non-significant decrease in the mean percentage of the vascular area and a statistically non-significant increase in the percentage of inflammatory cells when a PPD ≥9 mm was recorded.

### 4.5. Plaque

The percentage of the vascular area was statistically significantly higher in the no plaque group than in the plaque group. These data are difficult to explain considering the multifactorial etiology of GPIII–IVC [1]. The concomitant decrease in the percentage of inflammatory cells was explained by Sreedevi et al. [24]. In fact, they reported that suppression of the vascular inflammatory reaction could be linked to an impairment in the defense mechanisms and more susceptibility to plaque infection. It means that a higher vascular area in these kinds of susceptible patients could not be related to a relevant quantity of plaque at a level site. Moreover, a non-statistically significant augmentation of the percentage of inflammatory infiltrates was appreciated in the site with no plaque.

### 4.6. Pus

Considering the presence/absence of pus, no significant differences were observed. However, this factor should be investigated more.

Taking the statistical analysis into consideration, even if the above-considered factors are often commonly related to the worsening of periodontal status, the most significant one is smoking. Its effect contributes to the histopathological alteration, possibly worsening the clinical periodontal condition.

## 5. Conclusions

It is known that during gingival inflammation, inflammatory cells represent an increasing part within the connective tissue [8,9] and inflammation is associated with the activation of pathological angiogenesis and a high number of newly formed blood vessels [6,11].

In this study, we did not compare the percentage of the vascular area and inflammatory cells with healthy patients, but we evaluated how some factors (smoking, age, PPD, plaque) influenced these percentages in GPIII–IVC patients.

Within the limitations of the present study—the sample size—our results show a significant decrease in the percentage of the vascular area in association with smoking, age, and plaque and of inflammatory cell percentage in association with gender and smoking. The most significant effect is related to smoking in patients with a similar periodontal condition. For the future, new Randomized Clinical Trials – RCTs will be needed to verify whether smoking is a key factor in inflammatory and vascular periodontal alteration.

## Figures and Tables

**Table 1:** Clinical characteristics of patients (total mouth).

	Mean (sd)
PPD (mm)	3.46 (1.79)
CAL loss (mm)	4.03 (1.84)
Gingival recession (≠ 0)	2.39 (1.73)
	%
Gingival recession = 0	74.6
Bleeding on probing	49.0

**Table 2:** Mean percentage [and 95% CI] of inflammatory infiltrated and vascular area. * Raw log-means were estimated weighting for the inverse of variance within a patient and then transformed through exponential function. Only p-values of variables selected through backward regression models are reported.

	Mean Percentage of Inflammatory Infiltrates	Mean Percentage of Vascular Area
Overall Raw Weighted Mean *	4.38%	0.24%
Gender Female Male *p*-value	6.29% [3.21; 12.30] 2.28% [0.86; 6.04] *0.020*	0.47% [0.22; 0.93] 0.58% [0.22; 1.54] -
Age (median) <51 years old ≥51 years old *p*-value	3.21% [1.16; 8.88] 4.46% [2.54; 7.82] -	0.60% [0.22; 1.65] 0.46% [0.23; 0.86] *0.006*
Smoking habit Non-smokers Smokers *p*-value	4.56% [2.06; 10.09] 3.14% [1.44; 6.85] *0.048*	0.55% [0.32; 1.31] 0.41% [0.16; 1.06] *0.008*
PPD (median) <9 ≥9 *p*-value	3.61% [1.66; 7.85] 3.97% [1.84; 8.58] -	0.55% [0.28; 1.07] 0.59% [0.18; 1.31] -
Plaque Yes No *p*-value	3.01% [2.02; 4.49] 4.76% [1.53; 14.77] *0.14*	0.36% [0.17; 0.77] 0.74% [0.30; 1.77] *0.0005*
Pus Yes No *p*-value	3.67% [1.29; 10.42] 3.90% [2.00; 7.61] -	0.51% [0.12; 2.23] 0.53% [0.39; 0.71] -

## References

[1] Meyle J., Chapple I. (2015). Molecular aspects of the pathogenesis of periodontitis. Periodontol. 2000.

[2] Tonetti M.S., Greenwell H., Kornman K.S. (2018). Staging and grading of periodontitis: Framework and proposal of a new classification and case definition. J. Clin. Periodont..

[3] Pihlstrom B.L., Michalowicz B.S., Johnson N.W. (2005). Periodontal diseases. Lancet.

[4] Caton J.C., Armitage G., Berglundh T., Chapple I.L.C., Jepsen S., Kornman K.S., Mealey B.L., Papapanou P.N., Sanz M., Tonetti M. (2018). A new classification scheme for periodontal and peri-implant diseases and conditions—Introduction and key changes from the 1999 classification. J. Clin. Periodont..

[5] Cornelini R., Artese L., Rubini C., Fioroni M., Ferrero G., Santinelli A., Piattelli A. (2001). Vascular endothelial growth factor and microvessel density around healthy and failing dental implants. Int. J. Oral Maxillofac. Implants.

[6] Kasprzak A., Surdacka A., Tomczak M., Konkol M. (2013). Role of high endothelial postcapillary venules and selected adhesion molecules in periodontal diseases: A review. J. Periodontal. Res..

[7] Borsani E., Salgarello S., Mensi M., Boninsegna R., Stacchiotti A., Rezzani R., Sapelli P., Bianchi R., Rodella L.F. (2005). Histochemical and immunohistochemical evaluation of gingival collagen and metalloproteinases in peri-implantitis. Acta Histochem..

[8] Buffoli B., Dalessandri M., Favero G., Mensi M., Dalessandri D., di Rosario F., Stacchi C., Rezzani R., Salgarello S., Rodella L.F. (2014). AQP1 expression in human gingiva and its correlation with periodontal and peri-implant tissue alterations. Acta Histochem..

[9] Page R.C., Schroeder H.E. (1976). Pathogenesis of inflammatory periodontal disease. A summary of current work. Lab. Investig..

[10] Younes R., Ghorra C., Khalife S., Igondjo-Tchen-Changotade S., Yousfi M., Willig C., Senni K., Godeau G., Naaman N. (2009). Pertinent cell population to characterize periodontal disease. Tissue Cell.

[11] Vladau M., Cimpean A.M., Balica R.A., Jitariu A.A., Popovici R.A., Raica M. (2016). VEGF/VEGFR2 Axis in Periodontal Disease Progression and Angiogenesis: Basic Approach for a New Therapeutic Strategy. In Vivo.

[12] Know V., Caton J.C. (2007). Commentary: Prognosis revisited a system for assigning periodontal prognosis. J. Periodontol..

[13] Preshaw P.M., Henne K., Taylor J.J., Valentine R.A., Conrads G. (2017). Age-related changes in immune function (immune senescence) in caries and periodontal diseases: A systematic review. J. Clin. Periodontol..

[14] Meyer-Bäumer A., Pritsch M., Cosgarea R., el Sayed N., Kim T.S., Eickholz P., Pretzl B. (2012). Prognostic value of the periodontal risk assessment in patients with aggressive periodontitis. J. Clin. Periodontol..

[15] Tomasi C., Leyland A.H., Wennström J.L. (2007). Factors influencing the outcome of non-surgical periodontal treatment: A multilevel approach. J. Clin. Periodontol..

[16] Lang N.P., Tonetti M.S. (2003). Periodontal risk assessment (PRA) for patients in supportive periodontal therapy (SPT). Oral Health Prev. Dent..

[17] Jepsen S., Caton J.G., Albandar J.M., Bissada N.F., Bouchard P., Cortellini P., Demirel K., de Sanctis M., Ercoli C., Fan J. (2018). Periodontal manifestations of systemic diseases and developmental and acquired conditions: Consensus report of workgroup 3 of the 2017 World Workshop on the Classification of Periodontal and Peri-Implant Diseases and Conditions. J. Periodontol..

[18] Shin Y.J., Choung H.W., Lee J.H., Rhyu I.C., Kim H.D. (2019). Association of periodontitis with Oral Cancer: A Case-Control Study. J. Dent. Res..

[19] Malinowski B., Węsierska A., Zalewska K., Sokołowska M.M., Bursiewicz W., Socha M., Ozorowski M., Pawlak-Osińska K., Wiciński M. (2019). The role of Tannerella forsythia and Porphyromonas gingivalis in pathogenesis of esophageal cancer. Infect. Agent. Cancer.

[20] Shiau H.J., Reynolds M.A. (2010). Sex differences in destructive periodontal disease: Exploring the biologic basis. J. Periodontol..

[21] Prakash P., Rath S., Mukherjee M., Malik A., Boruah D., Sahoo N.K., Dutta V. (2014). Comparative evaluation of the marginal gingival epithelium in smokers and nonsmokers: A histomorphometric and immunohistochemical study. Int. J. Periodontics Restor. Dent..

[22] Calsina G., Ramón J.M., Echeverría J.J. (2002). Effects of smoking on periodontal tissues. J. Clin. Periodontol..

[23] Lindhe J., Lang N.P., Karring T., Berglundh T., Giannobile W.V. (2008). Clinical Periodontology and Implant Dentistry.

[24] Sreedevi M., Ramesh A., Dwarakanath C. (2012). Periodontal status in smokers and nonsmokers: A clinical, microbiological, and histopathological study. Int. J. Dent..

